# Differences in ultrasonographic features and diagnostic value of biopsy between tuberculous lymphadenitis and reactive lymphadenopathy

**DOI:** 10.1097/MD.0000000000049848

**Published:** 2026-07-24

**Authors:** Jun Peng, Zengpeng Chi, Jun Zhang, Weifei Li, Juanjuan Ren, Yajun Bi, Wenjing Lu, Meijia Hao

**Affiliations:** a Wuhan Tuberculosis Prevention and Control Institute, Wuhan Pulmonary Hospital, Wuhan, Hubei, China; b Department of Ultrasound, Third People’s Hospital of Hubei Province, Jianghan University, Wuhan, Hubei, China.

**Keywords:** core needle biopsy, diagnostic performance, fine-needle aspiration biopsy, reactive lymphadenopathy, scoring system, tuberculous lymphadenitis, ultrasound

## Abstract

This study aimed to investigate the differences in ultrasonographic features between tuberculous lymphadenitis (TBL) and reactive lymphadenopathy and evaluate the diagnostic value of biopsy techniques. A retrospective analysis was conducted on 225 cases of superficial lymph node lesions confirmed by pathology or microbiology between February 2022 and February 2024, including 128 cases of TBL and 97 cases of reactive lymphadenopathy. Ultrasonographic characteristics were compared between the 2 groups, and diagnostic performance was evaluated using pathological results as the gold standard. A multivariate logistic regression model was used to establish an ultrasound scoring system, and receiver operating characteristic curve performance was analyzed. The diagnostic accuracy and safety of fine-needle aspiration biopsy (FNAB) and core needle biopsy (CNB) were compared, and the benefits of biopsy were assessed based on ultrasound risk stratification. TBL cases more frequently exhibited round lymph nodes (S/L ≥ 0.5), liquefactive necrosis, loss of the hilum, calcification, and peripheral/mixed blood flow patterns (all *P* < .01). Liquefactive necrosis had the highest odds ratio (odds ratio = 14.47). Multivariate analysis identified these features as independent predictors. A 6-point ultrasound scoring system (≥3 points defined as high risk) achieved an area under the curve of 0.901, sensitivity of 82.0%, and specificity of 86.6%. CNB showed higher specimen adequacy and diagnostic accuracy compared to FNAB (*P* < .01), with no significant difference in complication rates. The positive biopsy rate was significantly higher in the high-risk group than in the low-risk group (95.9% vs 85.7%). Liquefactive necrosis, loss of the lymphatic hilum, and calcification are characteristic ultrasonographic features of tuberculous lymphadenitis. The ultrasound scoring system effectively improves the diagnostic accuracy of TBL. CNB offers advantages over FNAB in terms of sample adequacy and diagnostic efficiency. Ultrasound-based risk stratification can guide biopsy decisions and enhance overall diagnostic value. Different biopsy techniques may be appropriate under different clinical scenarios, and ultrasound-based risk stratification may help optimize individualized biopsy decision-making.

## 1. Introduction

Superficial lymphadenopathy is a common clinical sign shared by a variety of diseases, with complex etiologies that make differential diagnosis a persistent challenge in clinical practice.^[1]^ Among the various causes, infectious diseases play a major role. Tuberculous lymphadenitis (TBL), the most frequent form of extrapulmonary tuberculosis, remains highly prevalent worldwide.^[2]^ In contrast, reactive lymphadenopathy (RL) is typically caused by nonspecific infections or systemic inflammation of adjacent tissues and represents one of the most common benign conditions.^[3]^ Although both present as lymph node enlargement, their treatment strategies and prognoses differ markedly: TBL requires prolonged and standardized antituberculosis therapy, while RL usually resolves after management of the underlying condition or short-term anti-infective treatment.^[4]^ Therefore, early and accurate differentiation between TBL and RL is crucial for initiating appropriate therapy, avoiding antibiotic misuse, and preventing the dissemination of tuberculosis.^[5]^

High-frequency ultrasonography, characterized by its noninvasive, convenient, real-time, and high-resolution nature, has become the preferred imaging modality for assessing superficial lymph nodes.^[6]^ It allows for clear visualization of lymph node morphology (such as size, shape, and margins) and internal architecture (including the hilum and cortical-medullary structure), while color doppler imaging enables evaluation of vascular perfusion patterns.^[7]^ Previous studies have suggested that TBL exhibits certain characteristic ultrasonographic features, such as a round shape, loss of the fatty hilum, and internal liquefactive necrosis – findings that correspond to granulomatous inflammation with caseous necrosis on histopathology.^[8]^ However, these features are not specific to TBL, and considerable overlap exists with RL, resulting in limited diagnostic specificity and a degree of subjectivity when relying solely on ultrasound.^[9]^

When ultrasonography alone cannot yield a definitive diagnosis, ultrasound-guided biopsy becomes an essential technique for obtaining pathological confirmation. Currently, fine-needle aspiration biopsy (FNAB) and core needle biopsy (CNB) are the 2 primary approaches used in clinical practice. FNAB is simple and minimally invasive, but its diagnostic accuracy is highly operator-dependent and may be limited in detecting granulomatous inflammation due to the lack of preserved tissue architecture. In contrast, CNB retrieves core tissue samples that maintain microstructural integrity, facilitating histopathological evaluation and special staining, and theoretically offers superior diagnostic efficacy. However, comparative studies assessing these 2 methods in distinguishing TBL from RL remain limited.^[10]^

Given this context, the present single-center retrospective case-control study aims to systematically analyze and compare the ultrasonographic features of TBL and RL, identifying independent diagnostic predictors. Based on these findings, we propose to develop a simple and practical ultrasound scoring system to quantify diagnostic risk. Furthermore, we evaluate the diagnostic performance and safety of FNAB and CNB in real-world settings. Ultimately, our goal is to establish an evidence-based framework for ultrasound-guided risk stratification that informs biopsy decisions and integrates noninvasive imaging with invasive pathology for a precise diagnostic pathway.

## 2. Materials and methods

### 2.1. Study design

This study was approved by the Ethics Committee of Wuhan Pulmonary Hospital. This study was designed as a single-center retrospective case-control study. A total of 225 patients presenting with superficial lymphadenopathy who underwent ultrasonography and were subsequently confirmed by biopsy or surgical pathology at our hospital between February 2022 and February 2024 were included. Based on predefined inclusion and exclusion criteria, cases were screened and categorized into 2 groups according to final pathological or microbiological diagnoses: the tuberculous lymphadenitis (TBL) group and the RL group.

The TBL group included patients with histopathological evidence of tuberculous lesions or those with positive results on acid-fast staining, mycobacterial culture, or molecular tuberculosis tests (such as GeneXpert or TB-DNA). The RL group included patients whose pathology demonstrated reactive hyperplasia or nonspecific inflammation, and whose tuberculosis-related tests were negative. Among the total cohort, 128 cases (56.9%) were classified as TBL and 97 cases (43.1%) as RL. Only patients with pathological or microbiological confirmation were included in this study to ensure diagnostic accuracy and consistency of the reference standard. Patients diagnosed solely based on clinical follow-up without biopsy confirmation were excluded from analysis.

### 2.2. Inclusion and exclusion criteria

#### 2.2.1. Inclusion criteria

① Patients who underwent ultrasonography and ultrasound-guided biopsy or surgical excision for superficial lymphadenopathy at our hospital between February 2022 and February 2024;
② Availability of complete clinical data, ultrasonographic images, and confirmed pathological or microbiological results;
③ Final diagnosis of tuberculous lymphadenitis or RL based on pathology or microbiology;
④ First-time diagnosis or detection of lymph node lesions at the site, without prior targeted treatment;
⑤ Sufficient data quality, with analyzable imaging and pathological findings.

#### 2.2.2. Exclusion criteria

① Cases with pathological or final clinical diagnoses of other diseases (e.g., metastatic malignancy, lymphoma, sarcoidosis, cat-scratch disease, or Kikuchi disease);
② Incomplete or poor-quality ultrasonographic or pathological images insufficient for analysis;
③ Inadequate biopsy samples, indeterminate diagnostic outcomes, or sampling errors;
④ Patients who had received systemic antituberculosis or long-term anti-infective therapy before biopsy, potentially affecting imaging or pathological characteristics;
⑤ Duplicate cases due to repeated visits or multiple sampling from the same patient;
⑥ Incomplete clinical or pathological follow-up data, precluding confirmation of the final diagnosis.

### 2.3. Ultrasonographic examination methods and observational indicators

All patients underwent examination using the same model of color Doppler ultrasound diagnostic system equipped with a high-frequency linear array probe (frequency range: 7–15 MHz). Patients were positioned supine or in another suitable posture to ensure full exposure of the examination area. Scanning was performed longitudinally and transversely across the lesion site (neck, axilla, or groin) to observe and record the number, size, morphology, and internal structural characteristics of the affected lymph nodes. For each lesion, both the long and short axes were measured, and the short-to-long axis ratio (S/L) was calculated.

The main ultrasonographic parameters analyzed included:

① Shape and size: Measurement of the long and short diameters and calculation of the S/L ratio; nodes with S/L < 0.5 were defined as *oval*, and those with S/L ≥ 0.5 as *round*;
② Margins and capsule: Evaluation of margin clarity and the presence of capsular interruption or adjacent tissue invasion;
③ Internal echogenicity: Classification as homogeneous or heterogeneous hypoechoic, with attention to mixed echogenic regions;
④ Hilum structure: Assessment of whether the central hilum was distinct, displaced, or absent;
⑤ Liquefactive necrosis: Identification of central or eccentric anechoic areas with posterior acoustic enhancement;
⑥ Calcification: Observation of fine punctate, patchy, or coarse calcified foci;
⑦ Fusion and perinodal changes: Detection of multiple confluent lymph nodes forming clusters or alterations in surrounding soft tissue echogenicity.
⑧ Blood flow signals (color doppler flow imaging): Documentation of vascular distribution patterns (hilar, peripheral, mixed, or absent) and grading of vascularity according to the Adler 0–III classification.

All sonographic data were independently reviewed by 2 physicians holding intermediate or senior professional titles, each with over 5 years of experience in superficial ultrasonography, under blinded conditions. In cases of discrepancy, a third senior radiologist adjudicated to determine the final interpretation. In selected cases, elastography or superb microvascular imaging was additionally performed to supplement the evaluation of tissue stiffness scores and microvascular distribution patterns.

### 2.4. Biopsy procedure

All patients underwent ultrasound-guided biopsy procedures performed in real time by experienced sonographers. Depending on the lesion’s location, size, and depth, an appropriate biopsy technique was selected: for smaller lesions or those adjacent to vital blood vessels or nerves, FNAB was performed using 22 to 25G needles; for larger lesions or cases requiring preservation of tissue architecture, CNB was performed using 14 to 18G automatic cutting biopsy needles. The selection of FNAB or CNB was based on comprehensive clinical judgment, including lesion size, anatomical location, vascular proximity, procedural safety, and the anticipated need for preserved tissue architecture, rather than randomized allocation.

Before the procedure, a detailed assessment was made of the lesion’s position, dimensions, vascular anatomy, and surrounding critical structures to determine the optimal puncture pathway. Routine skin disinfection and local infiltration anesthesia were administered. Under continuous real-time ultrasound guidance, the needle was advanced along the predetermined path to ensure accurate entry of the needle tip into the target lesion. For each patient, 2 to 3 samples were obtained to ensure specimen adequacy. After biopsy, local compression was applied for 5 to 10 minutes to achieve hemostasis, and patients were observed for possible complications such as bleeding, hematoma, or pain.

All obtained specimens were promptly sent to the pathology department for histological examination, including routine hematoxylin and eosin staining and, when necessary, special staining (such as acid-fast staining). In selected cases, additional *Mycobacterium* culture and molecular testing (e.g., TB-DNA, GeneXpert) were conducted to aid diagnosis. All biopsy procedures were successfully completed, with no severe complications observed.

### 2.5. Data collection

This study adopted a retrospective data collection method. Data were obtained from the hospital’s picture archiving and communication system, Electronic Medical Record system, and the pathology department database. Data collection was independently performed by 2 researchers using a unified standardized form and cross-checked by a third investigator to ensure accuracy and completeness. The collected data mainly included 3 categories:

Clinical data: Patient demographic information (sex, age), disease course, number of lesions (solitary/multiple), systemic symptoms (such as fever, night sweats, and weight loss), and laboratory inflammatory markers (erythrocyte sedimentation rate, C-reactive protein).
Ultrasonographic data: Based on the stored images in the picture archiving and communication system system, all standardized observation parameters were recorded as described in section 2.2.
Biopsy data: The biopsy method (FNAB/CNB), puncture site, specimen adequacy, complications, and final pathological and microbiological diagnostic results were recorded.

### 2.6. Sample size and statistical power analysis

This study was a retrospective case-control study. According to preliminary experimental results and literature reports, the incidence rates of the main ultrasonographic feature (liquefactive necrosis) in tuberculous and reactive lymph nodes were approximately 60% and 10%, respectively. Setting α = 0.05 (2-sided) and test power (1 − β) = 0.90, the required sample size per group was calculated to be approximately 85 cases using the 2-proportioncomparison formula. Considering a 10% rate of missing or incomplete data, the theoretical minimum total sample size was about 190 cases. In this study, a total of 225 patients were finally included, with 128 in the tuberculous group and 97 in the reactive group. The actual sample size exceeded the calculated requirement, satisfying the statistical power needed for the primary comparative indicators. Post hoc power analysis of the main outcome variable (liquefactive necrosis incidence) showed a test power of 0.98, indicating that the study sample size was sufficient to reliably reflect the differences in ultrasonographic features and biopsy diagnostic performance.

### 2.7. Statistical analysis

Data were analyzed using SPSS 26.0 and MedCalc 20.2. Continuous variables were tested for normality with the Shapiro–Wilk test. Normally distributed data were expressed as mean ± standard deviation (*x̄* ± SD) and compared with the independent-samples *t* test; non-normal data were expressed as median (interquartile range) and compared with the Mann–Whitney *U* test. Categorical variables were expressed as frequencies and percentages and compared using the χ^2^ test or Fisher’s exact test. Standardized differences (Std Diff) were used to assess baseline comparability.

Univariate analysis identified differences in ultrasonographic features between groups and calculated odds ratios with 95% confidence intervals. Variables with *P* < .05 were entered into multivariate logistic regression to determine independent predictors. An ultrasound scoring model was established based on regression coefficients, and its diagnostic performance was evaluated by receiver operating characteristic curve analysis, calculating area under the curve (AUC), optimal cutoff (Youden index), sensitivity, specificity (Sp), positive predictive value, negative predictive value, and likelihood ratios (positive likelihood ratio, negative likelihood ratio). AUC differences were compared using the DeLong test.

Specimen adequacy, diagnostic accuracy, and complication rates between FNAB and CNB were compared with the χ^2^ test, while diagnostic performance differences were assessed by the *Z*-test or AUC comparison. Biopsy positivity rates between ultrasound risk groups (high vs low) were compared by proportion analysis. All tests were 2-tailed, with *P* < .05 considered statistically significant.

## 3. Results

### 3.1. Baseline characteristics and lesion distribution

As shown in Table 1, there were no statistically significant differences between the TBL and RL groups in terms of sex, age, disease duration, number of lesions, systemic symptoms, erythrocyte sedimentation rate, C-reactive protein levels, or lymph node distribution (cervical, axillary, or inguinal; all *P* > .05). These findings indicate that the 2 groups were comparable with well-balanced baseline characteristics.

**Table 1:** Baseline clinical characteristics and distribution of lesion sites between the two groups (n = 225).

Variable	Tuberculous lymphadenitis group (n = 128)	Reactive lymphadenopathy group (n = 97)	Statistic	*P* value	Standardized difference (Std Diff)
Sex (male/female), n	71/57	46/51	*χ*^2^ = 0.86	.35	0.09
Age (yr, mean ± SD)	43.2 ± 13.6	41.9 ± 12.8	*t* = 0.69	.49	0.1
Disease duration (mo, median [IQR])	4.2 (2.0–6.8)	3.7 (1.5–6.2)	*Z* = 0.97	.33	0.11
Number of lesions (single/multiple), n	84/44	59/38	*χ*^2^ = 0.56	.45	0.07
Systemic symptoms (present/absent), n	32/96	19/78	*χ*^2^ = 0.78	.38	0.09
ESR (mm/h, mean ± SD)	29.4 ± 11.8	27.9 ± 10.6	*t* = 0.98	.33	0.13
CRP (mg/L, mean ± SD)	12.8 ± 6.3	11.7 ± 5.9	*t* = 1.12	.26	0.12
Cervical region, n (%)	97 (75.8)	75 (77.3)	*χ*^2^ = 0.06	.81	0.03
Axillary region, n (%)	18 (14.1)	16 (16.5)	*χ*^2^ = 0.25	.62	0.06
Inguinal region, n (%)	13 (10.1)	6 (6.2)	*χ*^2^ = 1.03	.31	0.12

CRP = C-reactive protein, ESR = erythrocyte sedimentation rate, IQR = interquartile range, SD = standard deviation.

### 3.2. Comparison of ultrasonographic features

Significant differences were observed between the 2 groups in key ultrasonographic characteristics (Table 2). The TBL group more frequently exhibited round lymph nodes (S/L ≥ 0.5), indistinct margins or capsular interruption, loss of the hilum, heterogeneous internal echogenicity, liquefactive necrosis, calcification, nodal fusion, perinodal changes, and peripheral or mixed vascular patterns (all *P* < .01). Among these features, liquefactive necrosis showed the largest absolute intergroup difference (50.9%) and the highest odds ratio (OR = 14.47).

**Table 2:** Comparison of key ultrasonographic features between tuberculous and reactive lymphadenopathy groups.

Ultrasonographic feature	Tuberculous group (n = 128)	Reactive group (n = 97)	*χ*^2^/*Z*	*P* value	Absolute difference (%)	Odds ratio (95% CI)
S/L ratio ≥ 0.5	88 (68.8)	37 (38.1)	21.94	<.001	30.7	3.57 (2.05–6.21)
Blurred margin/capsule interruption	54 (42.2)	18 (18.6)	12.97	<.001	23.6	3.21 (1.70–6.06)
Hilum structure absent	91 (71.1)	29 (29.9)	36.87	<.001	41.2	5.68 (3.18–10.14)
Internal echogenicity heterogeneous	103 (80.5)	43 (44.3)	27.98	<.001	36.2	5.00 (2.76–9.05)
Central or eccentric liquefaction	77 (60.2)	9 (9.3)	63.08	<.001	50.9	14.47 (6.69–31.35)
Calcification (any type)	45 (35.2)	7 (7.2)	24.89	<.001	28	7.17 (2.99–17.19)
Confluent/matted nodes	38 (29.7)	8 (8.2)	14.92	<.001	21.5	4.67 (2.01–10.82)
Surrounding tissue change	33 (25.8)	9 (9.3)	9.9	.002	16.5	3.35 (1.53–7.32)
Blood flow pattern (CDFI) – peripheral/mixed	85 (66.4)	32 (33.0)	24.83	<.001	33.4	4.08 (2.31–7.21)
Absent blood flow (Adler 0)	22 (17.2)	5 (5.2)	7.3	.007	12	3.77 (1.32–10.74)

CDFI = color doppler flow imaging, CI = confidence interval, S/L = short-to-long axis ratio.

### 3.3. Diagnostic performance of individual ultrasonographic features

Using pathological or microbiological results as the gold standard, the diagnostic performance of each ultrasonographic feature in differentiating TBL from RL is summarized in Table 3. Liquefactive necrosis demonstrated the highest specificity (90.7%) and positive likelihood ratio (6.47), with an AUC of 0.854. Loss of the lymphatic hilum also showed relatively high sensitivity (71.1%) and AUC (0.807). Although calcification exhibited high specificity (92.8%), its sensitivity was relatively low (35.2%).

**Table 3:** Diagnostic performance of single ultrasonographic features for differentiating tuberculous lymphadenitis from reactive lymphadenopathy (pathology/microbiology as gold standard).

Ultrasonographic feature	Optimal cutoff	AUC (95% CI)	Sensitivity (%)	Specificity (%)	PPV (%)	NPV (%)	LR+	LR^−^
S/L ratio	≥ 0.50	0.751 (0.686–0.816)	68.8	61.9	70.4	60.1	1.81	0.51
Liquefactive necrosis	Present/absent	0.854 (0.802–0.906)	60.2	90.7	89.5	63.5	6.47	0.44
Hilum structure disappearance	Present/absent	0.807 (0.745–0.869)	71.1	70.1	75.8	64.5	2.38	0.41
Internal heterogeneity	Present/absent	0.792 (0.724–0.860)	80.5	55.7	70.6	68.3	1.82	0.35
Calcification	Present/absent	0.731 (0.659–0.803)	35.2	92.8	86.5	51.7	4.89	0.7
Matted lymph nodes	Present/absent	0.701 (0.625–0.777)	29.7	91.8	82.6	50	3.63	0.77
Peripheral/mixed vascularity (CDFI)	Present/absent	0.776 (0.709–0.843)	66.4	67	72.6	60.3	2.01	0.5

AUC = area under the curve, CDFI = color doppler flow imaging, CI = confidence interval, LR− = negative likelihood ratio, LR+ = positive likelihood ratio, NPV = negative predictive value, PPV = positive predictive value, S/L = short-to-long axis ratio.

### 3.4. Multivariate analysis and construction of the ultrasound scoring system

Variables with *P* < .05 in the univariate analysis were entered into multivariate logistic regression. The results showed that liquefactive necrosis, loss of the lymphatic hilum, S/L ≥ 0.5, calcification, and peripheral/mixed blood flow were independent predictors for identifying TBL (Table 4). Based on their regression coefficients (β values), a simple 6-point ultrasound scoring system was developed: liquefactive necrosis = 2 points, loss of hilum = 1 point, S/L ≥ 0.5 = 1 point, calcification = 1 point, and peripheral/mixed blood flow = 1 point. Receiver operating characteristic curve analysis indicated that when the total score was ≥3 points, the system achieved the best cutoff value, with an AUC of 0.901, sensitivity of 82.0%, and specificity of 86.6% for differentiating TBL.

**Table 4:** Multivariate logistic regression analysis for differentiating tuberculous from reactive lymphadenopathy.

Variable	Regression coefficient (β)	Standard error (SE)	Adjusted OR (95% CI)	Wald *χ*^2^	*P* value
Liquefactive necrosis (present = 1)	2.36	0.47	10.59 (4.22–26.56)	25.08	<.001
Hilum disappearance (present = 1)	1.28	0.39	3.60 (1.69–7.65)	10.67	.001
S/L ratio ≥ 0.5	0.97	0.33	2.63 (1.37–5.05)	8.65	.003
Calcification (present = 1)	0.82	0.36	2.28 (1.12–4.65)	5.19	.023
Peripheral/mixed vascularity (CDFI) (present = 1)	0.64	0.31	1.89 (1.04–3.43)	4.27	.039
Constant	−3.02	0.66	–	–	<.001

CDFI = color doppler flow imaging, CI = confidence interval, OR = odds ratio, S/L = short-to-long axis ratio.

### 3.5. Comparison of diagnostic value between biopsy methods

As shown in Table 5, the CNB group demonstrated significantly higher specimen adequacy (97.5% vs 88.6%, *P* = .008) and diagnostic accuracy (95.0% vs 81.9%, *P* = .002) compared with the FNAB group. The complication rates were low in both groups and showed no significant difference (5.0% vs 3.8%, *P* = .67). Regarding the diagnosis of TBL (Table 6), CNB outperformed FNAB in sensitivity (90.3% vs 81.4%), specificity (89.7% vs 86.2%), and AUC (0.924 vs 0.884), with the differences being statistically significant (*P* = .032).

**Table 5:** Comparison of adequacy rate, diagnostic yield, and complication rate between FNAB and CNB.

Parameter	FNAB group (n = 105)	CNB group (n = 120)	*χ*^2^/*Z* value	*P* value
Specimen adequacy rate (%)	93 (88.6)	117 (97.5)	7.01	.008
Definitive diagnostic rate (%)	86 (81.9)	114 (95.0)	9.25	.002
Positive detection rate for tuberculosis (%)	59 (56.2)	69 (57.5)	0.03	.86
Complication rate (%)	4 (3.8)	6 (5.0)	0.18	.67
Hemorrhage/hematoma (cases)	2	4	–	–
Local pain (cases)	2	2	–	–

CNB = core needle biopsy, FNAB = fine-needle aspiration biopsy.

**Table 6:** Diagnostic performance of FNAB and CNB for detecting tuberculous lymphadenitis (pathology/microbiology as gold standard).

Biopsy method	Sensitivity Se (%)	Specificity Sp (%)	Positive predictive value PPV (%)	Negative predictive value NPV (%)	AUC (95% CI)	*P* value (vs FNAB)
FNAB	81.4	86.2	89.3	76.1	0.884 (0.827–0.941)	–
CNB	90.3	89.7	92.8	86.1	0.924 (0.879–0.968)	.032
Combined (FNAB + CNB)	86.2	88.1	91	82.2	0.906 (0.862–0.950)	–

AUC = area under the curve, CI = confidence interval, CNB = core needle biopsy, FNAB = fine-needle aspiration biopsy, NPV = negative predictive value, PPV = positive predictive value, Se = sensitivity, Sp = specificity.

### 3.6. Diagnostic yield of biopsy under ultrasound risk stratification

Using the ultrasound scoring system developed in this study for risk stratification (Table 7), among 142 high-risk patients (score ≥ 3), 121 were pathologically or microbiologically confirmed as TBL, and 116 were correctly diagnosed by biopsy, yielding a positive diagnostic rate of 95.9%. In contrast, among 83 low-risk patients (score < 3), only 7 were finally confirmed as TBL, with a biopsy positivity rate of 85.7%. These results indicate that performing biopsy in the high-risk group provides a markedly higher diagnostic yield.

**Table 7:** Diagnostic yield of biopsy in different ultrasound risk stratification groups.

Ultrasound risk category	Number of patients (n = 225)	Cases confirmed as tuberculosis	Positive biopsy rate for tuberculosis diagnosis (%)
High-risk group (score ≥ 3)	142	121	95.9% (116/121)
Low-risk group (score < 3)	83	7	85.7% (6/7)
Total	225	128	95.3% (122/128)

## 4. Discussion

This study systematically analyzed 225 pathologically confirmed cases of superficial lymphadenopathy to explore the ultrasonographic differences between tuberculous lymphadenitis (TBL) and RL and to evaluate the diagnostic value of different biopsy techniques.^[11]^ The results demonstrated that the 2 groups were comparable in baseline characteristics but showed significant differences in ultrasonographic features, providing important clinical evidence for differential diagnosis.

From an imaging perspective, the TBL group more frequently exhibited a round shape, indistinct margins, loss of the lymphatic hilum, heterogeneous internal echogenicity, liquefactive necrosis, calcification, nodal fusion, perinodal changes, and peripheral or mixed vascular patterns.^[7]^ These findings correspond closely to the pathological processes of tuberculous lymphadenitis. The round morphology may reflect the expansive granulomatous growth typical of tuberculosis; loss of the hilum corresponds to the destruction of normal lymph node architecture^[12]^; liquefactive necrosis represents the sonographic manifestation of caseous necrosis; calcification reflects the chronic phase of the disease; and peripheral vascular patterns align with the neovascular distribution seen in inflammatory processes.^[13]^ Among these features, liquefactive necrosis had the highest diagnostic value, with an odds ratio of 14.47, strongly associated with the characteristic caseous necrosis of TBL.^[14]^

In terms of diagnostic performance, liquefactive necrosis showed the highest specificity (90.7%) and positive likelihood ratio (6.47) among individual ultrasound features.^[14]^ However, the multivariate analysis-based ultrasound scoring system demonstrated superior diagnostic accuracy, achieving an AUC of 0.901, with sensitivity and specificity of 82.0% and 86.6%, respectively, at the optimal cutoff value.^[15]^ This system integrates multiple independent predictors, with the greatest weight assigned to liquefactive necrosis, underscoring its central diagnostic importance. The scoring system’s strengths lie in its objectivity and practicality, helping to standardize image interpretation across operators.^[5]^

Regarding biopsy techniques, this study found that CNB demonstrated advantages in specimen adequacy and diagnostic performance, particularly in cases requiring preserved tissue architecture and histopathological evaluation. However, FNAB remains clinically valuable because of its simplicity, minimal invasiveness, and procedural safety.^[10]^ This superiority likely stems from CNB’s ability to obtain intact tissue cores that preserve the lymph node’s microstructure, allowing for the identification of granulomas and special staining. Nevertheless, FNAB remains valuable due to its simplicity and minimal invasiveness.^[3]^ Therefore, biopsy method selection should be individualized based on lesion characteristics, procedural risk, and diagnostic requirements.

The study also highlighted the value of ultrasound-based risk stratification in guiding biopsy decisions. Results showed that in the high-risk group, the positive diagnostic rate of biopsy reached 95.9%, while the prevalence of TBL in the low-risk group was very low.^[16]^ This finding suggests that performing biopsy in high-risk patients yields substantial diagnostic benefit, whereas a more conservative approach may be appropriate for low-risk cases.^[17]^ Such stratified diagnostic strategies can optimize resource allocation and minimize unnecessary invasive procedures.

It should be noted that this study has certain limitations. As a single-center retrospective study, potential selection bias cannot be excluded. Although image interpretation was performed under blinded conditions, some degree of subjectivity remained. Additionally, lymphadenopathy of other etiologies was not included as a control. The allocation of FNAB and CNB was not randomized; thus, potential indication bias could not be completely avoided. Lesions with higher clinical suspicion or more complex imaging characteristics may have preferentially undergone CNB, which may partially influence the observed diagnostic superiority of CNB. Future multicenter prospective studies are warranted to validate these findings, explore the added value of novel ultrasound techniques, and develop more comprehensive predictive models. Because only patients who underwent biopsy or surgical excision were included, cases diagnosed solely through clinical follow-up were not analyzed, which may have introduced selection bias and reduced representation of clinically mild or typical cases. In addition, the ultrasound scoring system proposed in this study should be regarded as a preliminary predictive model derived from a single-center retrospective cohort. Further multicenter prospective studies with external validation are required to evaluate its robustness, reproducibility, and generalizability before widespread clinical application.

In conclusion, this study systematically elucidated the ultrasonographic distinctions between tuberculous and RL and established an ultrasound scoring system with excellent diagnostic performance. It also clarified the appropriate application scenarios for different biopsy techniques. Ultrasound-based risk stratification can effectively guide clinical decision-making, enabling more accurate and efficient diagnostic workflows and providing valuable reference for clinical practice. Different biopsy techniques may be appropriate under different clinical scenarios, and ultrasound-based risk stratification may help optimize individualized biopsy decision-making.

## Author contributions

**Conceptualization:** Jun Peng, Zengpeng Chi, Jun Zhang, Weifei Li, Juanjuan Ren, Yajun Bi, Wenjing Lu, Meijia Hao.

**Data curation:** Jun Peng, Zengpeng Chi, Jun Zhang, Weifei Li, Juanjuan Ren, Yajun Bi, Wenjing Lu, Meijia Hao.

**Formal analysis:** Jun Peng, Zengpeng Chi, Jun Zhang, Weifei Li, Juanjuan Ren, Yajun Bi, Wenjing Lu, Meijia Hao.

**Funding acquisition:** Jun Peng, Meijia Hao.

**Investigation:** Jun Peng, Meijia Hao.

**Writing – original draft:** Jun Peng.

**Writing – review & editing:** Meijia Hao.
